# Addressing the Licensed Doctor Maldistribution in China: A Demand-And-Supply Perspective

**DOI:** 10.3390/ijerph16101753

**Published:** 2019-05-17

**Authors:** Bin Zhu, Chih-Wei Hsieh, Ying Mao

**Affiliations:** 1 School of Public Policy and Administration, Xi’an Jiaotong University, 28 Xianning West Road, Xi’an 710049, China; binzhu2-c@my.cityu.edu.hk; 2 Department of Public Policy, City University of Hong Kong, Tat Chee Avenue, Kowloon, Hong Kong, China; cwhsieh@cityu.edu.hk

**Keywords:** health workforce, supply and distribution, physicians, dentists, general practitioners, China

## Abstract

*Background*: The maldistribution of licensed doctors is one of the major challenges faced by the Chinese health sector. However, this subject remains underexplored, as the underlying causes of licensed doctor distribution have not been fully mapped out. To fill the research void, this study theoretically modeled and empirically measured various determinants of licensed doctor distribution from both the supply and demand sides while taking the spillover effect between the adjacent geographical units into consideration. *Methods*: The theory of demand and supply is adopted to construct a research framework so as to explain the imbalance in the licensed doctor distribution. Both direct effects and spillover effects of the supply-side factors and demand-side factors are empirically measured with the spatial panel econometric models. *Results*: The health service demand was found, as expected, to be the major driving force of the licensed doctor distribution across the nation. That is, the increase in health services demands in a province could significantly help one unit attract licensed doctors from adjacent units. Unexpectedly but intriguingly, the medical education capacity showed a relatively limited effect on increasing the licensed doctor density in local units compared with its spillover effect on neighboring units. In addition, government and social health expenditures played different roles in the health labor market, the former being more effective in increasing the stock of clinicians and public health doctors, the latter doing better in attracting dentists and general practitioners. *Conclusions*: The results provide directions for Chinese policy makers to formulate more effective policies, including a series of measures to boost the licensed doctor stock in disadvantaged areas, such as the increase of government or social health expenditures, more quotas for medical universities, and the prevention of a brain drain of licensed doctors.

## 1. Introduction

Health manpower maldistribution has long been a major challenge faced by the health sectors around the globe, with most health workers concentrated in the developed areas [1,2]. This pattern contributes greatly to health inequalities, which limits the health services accessibility in rural and remote areas. As the world’s most populous nation, China has the world’s largest troop of health manpower in a vast geographical area and therefore provides an exemplary case of a country facing health manpower maldistribution challenges [3]. Understanding the status of this large share of global human resources for health is of vital importance.

What factors may contribute to regional differences in the health manpower distribution? Many studies have tried to study health manpower maldistribution from an individual perspective [4]. For example, studies of job satisfaction, burnout, and motivation help explain the turnover behavior of health workers in rural and remote areas [5,6], while studies of medical students have shown how socio-economic characteristics and rural early exposure programs affect medical students’ intentions to choose rural medical work after graduation [7,8,9]. These studies explain how individual choices contribute to the health manpower maldistribution.

As an important part of the labor market, the distribution of health manpower is not only a function of health workers’ self-determination but also a “product” of macro-regional factors. At the macro-regional level, there is no widely used model for understanding the health manpower availability in a certain area. Scholz, Graf von der Schulenburg, and Greiner [10] found significant effects of demand/need factors (population morbidity/financial incentives) on health manpower in Germany. Laurence and Karnon [11] modeled the influence of population size, health needs, and service utilization rates on the health manpower supply in Australia. Murphy et al. [12] simulated how various factors affect the supply of health manpower in different scenarios in Jamaica. However, the generalizability of their findings to other countries is limited.

It is evident that why regional disparities exist in the distribution of health manpower is largely unanswered by existing studies. More studies are needed to explore the determinants of the health manpower distribution. To fill the research void, this study aims to model and measure the determinants of the licensed doctor distribution in China based on the theory of supply and demand. The research findings will serve as a basis for balancing the licensed doctor distribution in China. Licensed doctors are the most important component of health manpower in China due to their direct, significant role in healthcare delivery. In China, licensed doctors are referring to doctors with the certificate of (assistant) medical practitioner who are engaged in medical practice, exclusive of those in managerial positions [13]. They can be further divided into clinicians, traditional Chinese medicine (TCM) doctors, dentists, public health doctors and general practitioners (GPs). Therefore, the research contents of this study are: (1) To test and compare the effect of supply-side factors on the distribution of different subtypes of licensed doctors, (2) to test and compare the effect of demand-side factors on the distribution of different subtypes of licensed doctors, and (3) to showcase and compare the spillover effects of supply-side and demand-side factors to adjacent units on the distribution of different subtypes of licensed doctors.

This study represents one of the first attempts to interpret the licensed doctor distribution with supply and demand theory. The supply and demand theory has been widely employed to elucidate the dynamics of the labor market but has rarely been applied to the health field [14,15]. This study builds a research framework on the supply and demand theory so as to explain the imbalances of health manpower distribution. This research framework also has the merit of explaining the health manpower distribution in other contexts similar to China’s. More importantly, this study refines the supply and demand theory to adapt it for explaining the licensed doctor distribution within countries. As Anselin [16] pointed out, a given area would be more or less affected by its neighbors, but spatial spillovers between administrative units have been overlooked by existing studies on health manpower distribution. Considering the increasingly close connections of Chinese provinces, this study reasonably suspects that spatial spillovers could exist in the distribution of licensed doctors at the provincial level. Thus, spillover effects between adjacent units are incorporated into the research model, providing a theoretical reference for further in-depth studies. In other words, the licensed doctor density in one provincial unit is not only explained by the direct effects of the within-province factors but also by the spillover effects of the factors in adjacent provincial units. Moreover, both direct effects and spillover effects are empirically measured with the spatial panel econometric models, which verify the rationality and validity of the proposed research model.

## 2. Methodology

### 2.1. Hypotheses

This study follows the strategy proposed by Zhu et al. to explore the determinants of the licensed doctor distribution based on the theory of supply and demand [4]. The theory of supply and demand has served as an important basis for understanding the dynamics of the labor market. According to Dussault and Vujicic [17], the differences in supply and demand help us understand the licensed doctor distribution both within and between countries. The demand of licensed doctors can be defined as the number of licensed doctors that the health facilities are willing to hire, the supply as the number of licensed doctors who want to work in health facilities [17]. “In a perfect health labor market, supply would equal demand and there would be no imbalances” [17] (p. 83). However, in reality, the stock of licensed doctors is neither just the supply nor demand of licensed doctors, but is determined by the interactions of supply and demand [18].

**Figure 1:**
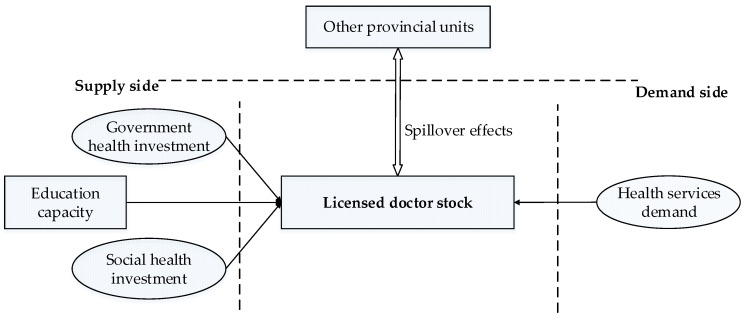
Research model of the determinants of licensed doctor distribution [4].

Figure 1 shows the research framework built to explain the determinants of the licensed doctor distribution in China, which can generally be divided into three parts: Demand-side factors, supply-side factors, and the spillover effects of other geographical units.

On the demand side, the population’s medical needs provide the fundamental impetus to hire licensed doctors. If there are no medical needs, licensed doctors would have no value [12]. However, it is important to differentiate the concepts of demand and need. The concept of need is normative—what ought to be, i.e., how many licensed doctors we should have [17]. It is determined by the population health status and demographic situation [19]. The concept of demand refers to how many licensed doctors the health system wants; it is only driven by people’s demand for health services. In general, the demand is invariably lower than the need for licensed doctors as there are always some unmet medical needs that are not reflected in the demand for health services on the account of multiple social and individual factors [4]. In other words, even though a large number of people have medical needs, the unmet needs cannot translate into the motivation of health facilities to recruit licensed doctors if they do not go to the hospitals or other health institutions. That is, the determination of the licensed doctor demand is driven by the people’s demand for health services rather than people’s medical needs [20]. The higher the health services demand, the more licensed doctors are needed. Therefore, we hypothesize the relationship between the health services demand and licensed doctor stock as follows:

**Hypothesis** **1.** *The health services demand in one provincial unit is related to the licensed doctor stock in the same provincial unit.*

In an ideal market, the licensed doctor stock is totally driven by the demand side, as there is no imbalance between supply and demand. In reality, the licensed doctor supply has normally failed to keep up with rising demand. Owing to the relatively undeveloped education system and growing medical needs, we reasonably believe that the licensed doctor availability in China is restricted, even to a large degree, by the supply side [4]. The supply chain of licensed doctors can be divided into two steps: Training and recruitment.

As the first stage, the education system serves as a pipeline from inexperienced students to skillful licensed doctors. Given that licensed doctors need to acquire professional job-related skills in order to provide quality services, medical education plays a crucial role in the supply of licensed doctors. More concretely, the education capacity determines the size of the pool of potential licensed doctors. Thus, this study hypothesizes that there is a positive relationship between the education capacity and licensed doctor stock as follows:

**Hypothesis** **2.** *Education capacity in one provincial unit is related to the licensed doctor stock in the same unit.*

Next, following the graduation of medical students, the recruitment of licensed doctors relies on the attraction and retention of health facilities. In China, medical institutions are classified into two groups, public health institutions (not-for-profit) and private health institutions (for-profit). In public health institutions, the number of employees is mainly determined by the budget allocated, as a significant portion of government health expenditures are assigned to health workers [21]. Therefore, those provincial units with greater financial resource allocations by the government are better positioned to recruit, retain, and compensate licensed doctors. For this reason, we postulate:

**Hypothesis** **3.** *Government health investment in one provincial unit is related to the licensed doctor stock in the same unit.*

In private health institutions, licensed doctor enrollment is mainly determined by the investment of social capital. As far as province units are concerned, greater social health investment indicates an influx of more licensed doctors, as it brings more employment opportunities in private health facilities in its train. In addition, the competition of public and private hospitals tends to offer better working conditions, thus attracting more licensed doctors working in the area. Therefore, this study proposes the following hypothesis about the relationship between the social health investment and the licensed doctor stock:

**Hypothesis** **4.** *Social health investment in one provincial unit is related to the licensed doctor stock in the same unit.*

In addition to the intra-regional factors, it is important to note that the spillover effects (the effects of within-province factors on adjacent provinces [22]) cannot be ignored when studying the licensed doctor distribution in China. The reason is that innumerable links exist between provincial units, resulting in the spillover effects of intra-regional factors to other units. For instance, the immigration of licensed doctors that results from wage differentials is the embodiment of the spillover effects between different geographical units.

Therefore, the licensed doctor stock in one provincial unit calls for explanation not only by the supply-side factors and demand-side factors in the local unit but also through its interactions with other provincial units. The stronger the supply-side and demand-side factors are in one area, the more likely licensed doctors in other areas are affected, and vice versa. For instance, if the government health investment in one unit is higher than in other units, licensed doctors from adjacent units are more likely to be attracted to it. Therefore, this study suggests the following hypothesis:

**Hypothesis** **5.** *The health service demand, education capacity, government, and social health investment in one provincial unit are related to the licensed doctor stock in adjacent units.*

### 2.2. Measurement of Variables

#### 2.2.1. Licensed Doctor Stock: Licensed Doctor Density

This study uses the licensed doctor density as the dependent variable. It is defined as the ratio of licensed doctor quantity to population size and conventionally calculated as the total licensed doctors per 1000 population (Formula (1)). It is widely used in the World Health Organization (WHO) reports, government statistical files, and academic research to evaluate the performance of a health system [23,24,25,26]. As different subtypes of licensed doctors play different roles in the operation of the healthcare system, different subtypes of licensed doctors will be discussed separately.

(1) $$Licensed\;doctor\;density=\frac{Licensed\;doctor\;number}{Population}\times 1000$$

#### 2.2.2. Health Service Demand: Outpatient Visits per Capita and Inpatient Visits per Capita

Health services are defined as a certain quantity or a combination of different services, such as diagnosis, observation, treatment, and intervention services, that are delivered by health institutions [27]. Health services can be roughly divided into outpatient services and inpatient services [28]. An outpatient means a patient who visits health institutions (hospital, clinic, etc.) but is not hospitalized overnight, while inpatients receive lodging in health institutions. As patients are only hospitalized overnight when they are extremely ill or have severe physical trauma, the outpatient and inpatient visits therefore reflect different aspects of health services demand. This study adopts the ratios of both outpatient and inpatients visits and population, i.e., outpatient visits per capita and inpatient visits per capita, to measure the health services demand, as higher per capita inpatient and outpatient visits reflect stronger health service demand.

#### 2.2.3. Education Capacity: Medical Graduate Density

Medical schools serve health institutions by pipelining trained medical graduates into the health manpower pool. Even though the number of medical graduates does not fully represent the medical education capacity in one unit, it directly determines the number of potential health workers. The more students the medical education system trains, the larger the potential labor force for the health industry. To maintain consistency with the dependent variable, this study adopts the medical graduate density, i.e., the ratio of the number of medical graduates to the population size, to represent the education capacity in a unit.

#### 2.2.4. Government and Social Health Investment: Government Health Expenditure per Capita and Social Health Expenditure per Capita

In the Chinese context, the health expenditure is divided into three parts: Government, social, and personal health expenditure. Government and social health expenditure correspond exactly to the government and social health investment. The government health expenditure represents the investment in the health care industry by all branches of governments, while the social health expenditure refers to the investment in the health care industry by all sectors of society other than the government [29]. Likewise, this study also adopts the ratio of health expenditure to the population, that is, the government health expenditure per capita and social health expenditure per capita, to represent the investment level of government and society, respectively.

A summary of all the variables and their codes and descriptions is shown in Table 1.

### 2.3. Data and Models

This study uses the provincial level year-end data in China from the period 2012−2016. Figure S1 displays the administrative divisions of China. Only the provincial administrative units in China mainland are included in this study. The data in this part were obtained from the China Statistical Yearbook (CSY), China Health Statistical Yearbook (CHSY), China Health and Family Planning Statistical Yearbook (SHFPSY), and China Education Statistical Yearbook (CESY). All the original data adopted in the study of determinants can be found in Supplementary material Table S1. The detailed description and resources of the variables are displayed in Table 2.

This study is based on the following model (Formula (2)) derived from the theoretical model in Section 2.1 and the measurement of the variables.

(2) $$\begin{array}{c} Ln{\left(LDD\right)}_{it}=\alpha +\beta_{1}ln{\left(OV\right)}_{it}+\beta_{2}ln{\left(IV\right)}_{it}+\beta_{3}ln{\left(GHE\right)}_{it}+\beta_{4}ln{\left(SHE\right)}_{it}+\beta_{5}ln{\left(MGD\right)}_{it}+\epsilon_{it}\\ i=1,2\ldots 31;\\ t=2012,\;2013,\;2014,\;2015,\;2016 \end{array}$$

The subscript *i* represents 31 province units (*i* = 1, 2, …, 31) and *t* denotes time *t* (*t* = 2012, 2013, 2014, 2015, 2016). α stands for the constant term and ϵ for the error term. It is important to make a note that this study uses the logarithmic transformation in the regression models to minimize the heteroskedasticity effect. This implies that the coefficient reflects the percentage change of dependent variables to the independent variables proportionately.

As the spillover effects of within-province factors on other units should be considered, the traditional regression models fail to measure the spillover effects between adjacent geographical units. This study, therefore, adopts the spatial panel econometric models so as to produce a more accurate estimation of model coefficients and spillover effects [30]. Spatial panel econometric models can be regarded as the extension of conventional regression models by adding information of adjacent units [31]. In general, the spatial panel econometric model includes three basic models: The Spatial Lag Panel Model (SLPM), the Spatial Error Panel Model (SEPM), and the Spatial Durbin Panel Model (SDPM) [32,33]. In order to avoid the estimation bias caused by the Ordinary Least Squares (OLS) estimation when spatial effects exist [16], the maximum likelihood method is adopted to estimate the coefficients of spatial panel econometric models.

SLPM (Formula (A1) in Appendix A) is mostly employed to analyze the spatial autocorrelation of the dependent variable, that is, whether the value of a unit is influenced by values of its neighboring regions [34]. The SEPM (Formula (A2) in Appendix A), however, is used most often to address the circumstance where the dependent variable is related to a group of variables as well as to the spatially autocorrelated error term. SDPM (Formula (A3) in Appendix A) is most useful when the dependent variable should be explained by the independent variables in both the local unit and adjacent geographical units [35]. Since SDPM exploits the complicated dependence structure between units, the effect of an explanatory variable change for a specific unit will affect the unit itself and potentially affect all adjacent units indirectly. The changes of variables in the neighboring units can further affect the specific unit through feedback. Hence, the effects of independent variables on the dependent variable in SDPM models can be further decomposed into direct and spillover effects. By definition, the direct effects refer to the influence of a unit change in an independent variable *x* in the geographical unit *i* on the health manpower density in the geographical unit *i,* averaged across all the geographical units. In comparison, the spillover effects (indirect effects) refer to the influence of a unit change in the independent variable *x* in all the geographical units excluding unit *i* on the health manpower density in the geographical unit *i*, averaged across all the geographical units. The total effects equal the sum of the average direct and average spillover effects and measure the impact of a unit change in the independent variable *x* in all the units on health manpower density in the geographical unit *i*, again averaged across all the geographical units [36].

**Figure 2:**
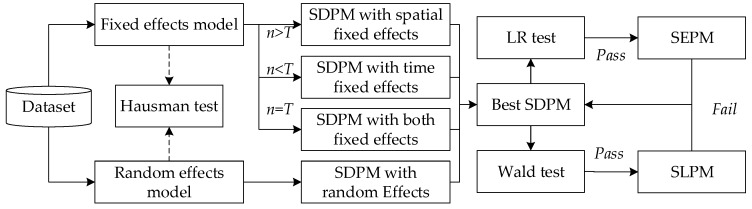
Model selection procedures of the spatial panel econometric models [37].

The procedures for the specification of spatial panel econometric models are shown in Figure 2. Following the strategy proposed by Elhorst et al. [38], researchers should start with the SDPM and test for alternatives. That is, we estimate the SDPM but would like to know if it is the best model for the specific data. However, as this study uses the panel data, the first task is to make a judgment between all the SDPM sub-models. Therefore, the model selection can be divided into two phases. First, we estimate all the SDPMs to test which is the best. Second, we run two tests to judge whether the best SDPM can be simplified into SLPM or SEPM [4].

In the first phase, the SDPM can be divided into a model with spatial-fixed effects (controlling the “space-specific, time-invariant” variables, which are excluded from the model), a model with time fixed effects (controlling “all time-specific, space-invariant variables,” which are excluded from the model), a model with spatial and time fixed effects (controlling the above two), and a random effects model based on different assumptions [32]. According to Greene [39], we should choose the one-way fixed models (spatial-fixed or time-fixed) if the sample size *n* does not equal *T* compared with the two-way fixed model. More specifically, a model with spatial fixed effects for short panels (*n* > *T*) and a model with time fixed effects for long panels (*n* < *T*). Furthermore, the choice between models with fixed and random effects should be made based on the Hausman test [40].

In the second phase, the Wald test (H_0_: θ = 0) and LR (likelihood ratio) test (H_0_: *θ* = −ρβ) are successively used to test the alternatives of SDPM. It is easily shown that if θ = 0 and ρ ≠ 0, the SLPM is a better one, while if θ = −ρβ, the model is SEPM where errors are spatially correlated. But if the conditions *θ* = 0 and *θ* + ρ*β* = 0 are both rejected, the most fit one would be SDPM [41]. To fully capture the possible spatial correlations between adjacent units, both these spatial panel econometrics models will be estimated and compared in order to choose the model that can best illustrate the relationship between the dependent and independent variables.

### 2.4. Softwares

GeoDa 1.8.16 (Version 1.8.6116, the University of Chicago, Chicago, IL, USA) is employed to constitute the spatial weight matrix. The spatial panel econometric models are computed with the STATA 12.0 (Version 12.0, StataCorp, College Station, TX, USA) [36]

## 3. Results

### 3.1. Descriptive Statistics

**Figure 3:**
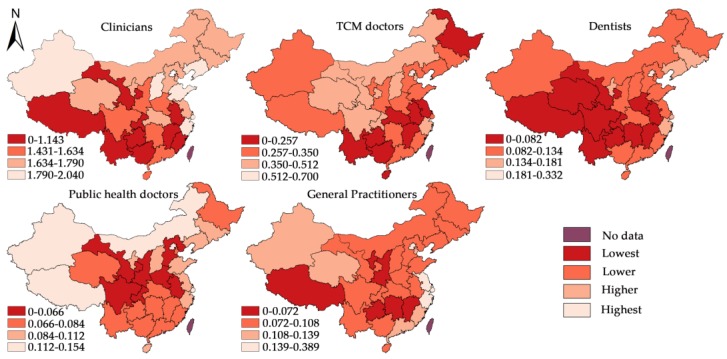
Average densities of different subtypes of licensed doctors during 2012–2016.

Table 3 reports the descriptive statistics of the main variables employed in the study of determinants. Totally, there are five dependent variables and five independent variables. Their relationships will be tested respectively with different spatial panel econometric models. Figure 3 displays the hierarchical maps of average densities of different subtypes of licensed doctors during 2012–2016. In each map, 21 provincial units are divided into four groups (Lowest, lower, higher and highest from dark red to light red) based on natural breaks methods, i.e., maximum variance between groups and minimum variance within groups. In general, the distribution of the five subtypes of licensed doctors displayed relatively different characteristics, while the units located in southwest China are suffering shortage for almost all subtypes of licensed doctors.

### 3.2. Empirical Results of Spatial Panel Econometric Models

Following the procedures specified in the methodology section (Figure 2), this study first estimated four SDPM models: The SDPM with spatial fixed effects, with time fixed effects, with spatial and time fixed effects, and with random effects. The model selection principles were then followed to choose the best model. In the context of this study, the SDPM with spatial fixed effects was preferred to the time fixed and both fixed models, as the dataset belonged to the short panel (*n* > *T*). Therefore, the Hausman test was conducted to make a choice between the SDPM with fixed effects and the random effect model. After the Hausman test, the LR and Wald tests were conducted successively to determine whether the SDPM model could be simplified. After this step, the best model was obtained. The empirical results of the spatial panel econometric models for different subtypes of licensed doctors can be found in supplementary material Table S2 (clinicians), Table S3 (TCM doctors), Table S4 (dentists), Table S5 (public health doctors), Table S6 (GPs), respectively.

The best spatial panel econometric models for different subtypes of licensed doctors are listed in Table 4. In this study, the SDPM was the best model in most cases, namely, those of clinicians, public health doctors, and dentists. As for TCM doctors, the SLPM with random effects was selected, as the results could not pass the Hausman and Wald tests. It is noteworthy that, based on the results of the Hausman, Wald, and LR tests, the density of general practitioners should not be estimated by spatial panel econometric models, and that the non-spatial panel model was shown to be a better one. Therefore, this study estimated the non-spatial model for general practitioners and conducted the Hausman test to make a choice between models with fixed effects and random effects.

After all the best models were identified, the relationship between dependent variables and independent variables (positive or negative, significant or not) were clear at a glance. However, the coefficients of variables in SDPM could not be explained directly as the effects of percentage variation in independent variables on dependent variables thanks to the unique characteristics of spatial panel econometric models, which have been mentioned in the methodology section. Thus, we decomposed the effects of the independent variables on the dependent variables into direct, spillover, and total effects based on the selected best models.

### 3.3. Decomposition of the Direct, Spillover and Total effects

Table 5 reports the direct effects of independent variables on the density of different subtypes of licensed doctors. The outpatient visits per capita played a huge role in driving the densities of almost all subtypes of licensed doctors, except dentists. A 1% increase of outpatient visits per capita corresponded to a 0.628% increase in clinician density. The coefficients could be as high as 0.959% and 0.756% for public health doctors and general practitioners, respectively. As for the TCM doctors, a 1% increase in the number of outpatient visits per capita was only associated with a 0.10% increase in the density of TCM doctors. All these estimates reached the 5% significance level.

Regarding the inpatient visits per capita, the estimated results showed a significant relationship between the inpatient visits per capita and the densities of clinicians, public health doctors and general practitioners, which were significant at the 0.01 significance level. A 1% increase in the inpatient visits per capita led to a 0.256% increase in the density of clinicians, 0.344% in public health doctor density and 0.703% in general practitioner density in the same unit.

The government and social health expenditure per capita had both similarities and differences in driving the densities of subtypes of licensed doctors. They were both significantly related to the TCM doctor density and general practitioner density. A 1% increase in government health expenditure in the local unit could lead to a 0.139% (0.158% by social health expenditure) increase in the TCM doctor density in the same unit. In contrast, a 1% increase of government and social health expenditure per capita in the local unit could respectively result in a 0.302% and 0.425% increase in the general practitioner density in the same unit. The government health expenditure also affected the clinician density (a 1% increase in expenditure corresponds to a 0.085% increase in the clinician density) and public health doctor density (a 1% increase in expenditure corresponds to a 0.260% increase in the public health doctor density). By comparison, a 1% increase in social expenditure per capita was also related to a 0.210% increase in the dentist density in one unit. As for the medical graduate density, only the density of clinicians was significantly correlated with the medical graduate density at the 0.1 significance level.

Table 6 reports the spillover effects of independent variables on the density of different subtypes of licensed doctors in adjacent units. As for the clinicians, the findings showed that the spillover effects of outpatient and inpatient visits per capita and the medical graduate density were both significant for clinicians. It was estimated that a 1% increase in outpatient and inpatient visits per capita in one provincial unit would lead to a 0.461% and 0.306% decrease in the clinician density in adjacent units, while a 1% increase in the medical graduate density could bring a 0.090% increase in the clinician density in adjacent units. Concerning the TCM doctors, the spillover effects of government and social health expenditure were positive and significant. A 1% increase in government health expenditure per capita in the local provincial unit would on average lead to a 0.043% (0.050% by social health expenditure per capita) increase in the TCM doctor density in all the neighboring units. In the case of dentists, the 1% increase in social health expenditure per capita in the local unit could lead to a 0.144% increase in the dentist density in the neighboring units, which only reached the 0.1 significance level. In contrast, a 1% increase of the medical graduate density in the local unit could result in a 0.200% increase in the dentist density in the neighboring units, which was significant at the 0.01 significance level. Surprisingly, a 1% increase in inpatient visits per capita could result in a 0.489% decrease in the public health doctor density in adjacent units.

### 3.4. Comparison and Visualization of the Direct and Spillover Effects

**Figure 4:**
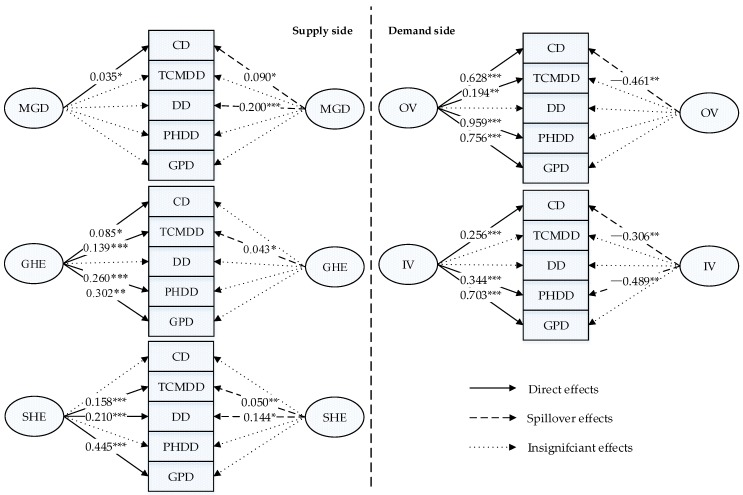
Summary of direct and spillover effects of supply-side and demand-side factors on the densities of different subtypes of licensed doctors. Note: *** *p* < 0.01, ** *p* < 0.05, * *p* < 0.1. CD = Clinician density, TCMDD = TCM doctor density; DD = Dentist density, PHDD = Phblic Health Doctor density; GPD = General practitioner density; OV = Outpatient Visits per capita; IV = Inpatients visits per capita, GHE = Government health expenditure per capita, SHE = Social health expenditure per capita, MGD = Ln Medical graduate density. The numbers in the figure are the coefficients of independent variables and dependent variables.

Figure 4 summarizes the direct (left side) and spillover effects (right side) of supply-side and demand-side factors, respectively, on the densities of different subtypes of licensed doctors. The findings confirmed the significant direct effects of both outpatient and inpatient health services per capita on the density of licensed doctors, indicating that the health services demand was pivotal in driving the licensed doctor density. The regression results suggested a significantly positive correlation between outpatient and inpatient health services per capita in one unit and the densities of clinicians, public health doctors, and GPs in the same unit, while this study failed to find a significant relationship between inpatient health services per capita and the density of TCM doctors.

As shown on the right side of Figure 3, a province’s increased health service demand adversely affected the densities of core types of licensed doctors in adjacent units, which implied that the increase in the health service demand in one province had a negative impact on its neighboring provinces. More specifically, if there were high medical needs in one unit, this not only accompanied higher licensed doctor density in the respective unit, but that unit was also more likely to attract the clinicians from adjacent units. Unexpectedly, the increase of inpatient health service demand could also help attract public health doctors from adjacent units.

This study considered the medical graduate density an important independent variable and then observed a significant relationship between it and the density of clinician density in the same unit. Intriguingly, the medical education capacity had relatively strong spillover effects on adjacent units compared to its direct effects. The results confirmed the positive and significant spillover effects of the medical education capacity on the densities of clinicians and dentists.

In addition, the government and social health expenditure played different roles in the health labor market. As for the direct effects, different subtypes of licensed doctors showed different sensitivities to government and social health expenditure. First, government and social health expenditure per capita in one unit were both potent predictors of the density of the TCM doctor density, and GP density. These practitioners provide health services directly and thus by their nature are a major concern of the government and target of social health expenditure. Second, some subtypes of health technicians were sensitive to the investment of only one type of health expenditure. That is, the densities of clinicians, public health doctors were only sensitive to government health expenditure, while the densities of dentists were only significantly related to the social health expenditure per capita. Regarding the spillover effects, the social health expenditure in one unit exerted much more pronounced spillover effects on adjacent units than the government health expenditure. An increase of the government health expenditure per capita in one unit only served to increase the density of TCM doctors in the adjacent units, but the increase of the social health expenditure per capita in one unit was associated with increases in the densities of TCM doctors and dentists in the adjacent units. This indicates that the social health expenditure investment in one unit could also benefit its neighbors more through spillover effects.

## 4. Discussion

On the basis of a five-year (2012–2016) panel dataset, this study empirically measured the determinants of densities of different subtypes of licensed doctors in China. The supply-side factors, demand-side factors, and spillovers jointly drive how licensed doctors are distributed. In general, the direct and spillover effects of all the independent variables achieved statistical significance for at least one subtype of licensed doctor, which confirms the rationality and validity of the theoretical model, while the coefficients for the effects of the independent variables on the densities of different subtypes of licensed doctors varied greatly.

On the demand side, the health service demand forcefully drove the licensed doctor density at the provincial level, while the outpatient and inpatient health service demand exerted relatively different influences, which also differed across subtypes of licensed doctors. Based on the empirical results of spatial panel econometric models, the densities of almost all the subtypes of licensed doctors were significantly related to the outpatient health service demand in the same units. It is not surprising that this study failed to find a significant relationship between inpatient health services per capita and the density of TCM doctors, which can be attributed to the relatively weak role of TCM doctors in inpatient services. That is, TCM doctors usually adopt non-surgical treatment methods, like herbal medicine, acupuncture, massage, and dietary therapy, for which patients do not need to stay in health institutions overnight. In addition, neither the outpatient nor inpatient health service demand is significantly correlated with the dentist density. This may result from the undifferentiated measurement of the health services demand. Due to data availability, this study only adopted total health service visits per capita rather than oral care service visits to predict the dentist density. However, dentists only provide a small proportion of health services and serve a smaller number of patients [42].

With regard to spillover effects, the uneven health service demand indicated in the results is likely to induce a brain drain of licensed doctors by siphoning off the health talents of surrounding regions. However, only the density of clinicians was significantly related to the outpatient and inpatient health service demand in adjacent units. This may also be attributed to the direct role of clinicians in the outpatient and inpatient service delivery. What outpatient and inpatient services have in common is that they directly connect patients and licensed doctors. The more important a subtype of licensed doctors is in the health service delivery, the more likely this subtype of licensed doctors is to be attracted to a high-demand area. Accordingly, only the attraction and enrollment of clinicians can help meet the health service demands in local units more efficiently.

These findings suggest that the government should take health service demand into the consideration of licensed doctor planning. At present, China only uses the licensed doctor density to determine manpower requirements and fails to consider the wide local variations in health service demand. More specifically, extreme health service demand differences could be observed at the provincial level ([3.007, 10.716] for the OVPA range and [0.047, 0.223] for the IVPA range, see the summary statistics in Table 2), indicating that the workload of licensed doctors differs across regions. Following WHO suggestions, the Workload Indicators of Staffing Need (WISN) is a complementary, systematic way to manage health manpower well [43]. It calculates health worker requirements based on the health workers’ workload, with activity (time) standards applied for each workload component. We believe that it holds promise for resolving the problems of allocating licensed doctors in China due to the significant role of the health service delivery in driving licensed doctor distribution. The finding indicates that a balanced licensed doctor distribution cannot be achieved only with efforts from the supply side. At first sight, the government is limited on the demand side, as one’s health services demand is seemingly difficult to manipulate. Yet, the population’s health services demand can be stimulated by means of health policies, like health insurance and medical assistance, as these medical needs have never been fully met [4]. Based on these findings, if the medical needs repressed by economic conditions can be better satisfied, licensed doctor densities will increase.

On the supply side, medical education benefits not only the local health system but also the surrounding regions. The spillover effects of education have been shown and explored by many existing studies [44]. This study for the first time proved the spillover effects of medical education on the recruitment of licensed doctors in adjacent units. Furthermore, the spillover effects of medical education on neighboring units are stronger among the core type of licensed doctors (i.e., clinicians). This may indicate the strong mobility of medical graduates. That is, it is common for medical students to choose a workplace in an adjacent provincial unit after graduation for personal reasons, like socioeconomic factors, family, or good career prospects [45]. However, investment in the health system requires time to achieve substantial progress [17]. On the one hand, it takes years to nurture medical students. In 2013, China launched a nationwide reform of medical education, called 5 + 3 (five-year undergraduate and three-year standardized residency training), to ensure education quality, which requires medical students to take additional time to formally enter the health labor market [3]. On the other hand, after employment, medical graduates would also need time to develop and master the skills necessary for the job [46]. These both require the education sector to plan ahead.

When it comes to health expenditure, this study showed a significant relationship between either the government or social health expenditure and the TCM doctor and general practitioner densities in a given unit. This finding echoes studies using the GNI per capita and government health expenditure to predict the density of health manpower [47,48,49] and fortifies the notion that the investment of financial resources can improve the accessibility of healthcare services [50]. However, different subtypes of licensed doctors showed different sensitivities to the government and social investment health. This seemingly surprising result, we suspect, may be attributed to the differing focus of the government and social capital and competition between public and proprietary hospitals in recruiting health workers. Solicitude for the common good is an essential character of the government health expenditure, causing it to be focused on the licensed doctor subtypes that are committed to the public welfare. For instance, public health doctors rely heavily on subsidies from the government as they provide non-profit public health services like establishing health records; screening for major diseases in elderly people; and providing health education and managing chronic non-communicable diseases [51,52]. In contrast, the social health expenditure tends to invest in dentists, who are essential in high-profit oral care services. Besides, due to the narrower supply channel of licensed doctors, competition is particularly keen in the recruitment of doctors. Therefore, proprietary hospitals may be disadvantaged in the attraction and retention of some subtypes of licensed doctors, such as clinicians. Tang et al. [53] found that many doctors in proprietary hospitals used to work in public hospitals, which reveals that proprietary hospitals may develop at the expense of public hospitals, especially when competing for the services of clinicians. Therefore, the total number of clinicians in a region may not increase with the growth of social health expenditure there, as it may only change clinicians’ place of practice. Precisely because the government and social health investment differ in their fundamental nature (the common good versus profitability), they play different but complementary roles in licensed doctor allocation [53,54]. Therefore, the path to higher coverage of licensed doctors requires the collaboration of the government and social health investment [4]. If only the government is involved, it is very likely that the health labor market will face a shortage of funds, while in the case of the exclusive involvement of social health expenditure, markets may fail due to the constrained reactions of the licensed doctor supply and demand to price signals in the health labor market [55].

This study also bears some limitations. This study chose only five independent variables to predict the density of health manpower at the provincial level due to data availability. We certainly believe that more socioeconomic factors should be considered, but they were not included in this study. Moreover, this study only considered the spillover effects of adjacent units. In fact, the spillovers can exert effects at greater distances due to political and economic factors. For instance, health workers are more likely to be attracted to the capital or larger cities no matter where they are in China.

## 5. Conclusions

Based on the Chinese context, this study tested the supply-side and demand-side factors and measured their indicators using the government data in five consecutive years (2012–2016). As validated by the results, the supply and demand theory works well in exploring the behaviors of licensed doctors at the macro level. Besides, the health service demand, medical education, and social health investment in one unit could significantly influence the licensed doctor stock in adjacent units. 

This study therefore provides a theoretical framework to understand the licensed doctor distribution, flow and brain drain between different regions in China and highlights an important research direction for further in-depth studies. The results provide evidence for Chinese policy makers to formulate more effective policies, including a series of measures for balancing the licensed doctor distribution in China: First, this study highlights that the health sector should proactively allocate licensed doctors through the health service demand. The low licensed doctor density in some units may result from the population’s inability to meet their medical needs. In those disadvantaged units, the government is supposed to take a stronger role to meet more of the population’s needs and strengthen the financial protection they have to avoid people falling into poverty because of illnesses. The more people’s needs are satisfied, the more power health institutions can obtain to attract and recruit licensed doctors. Second, due to the significant supporting role of medical education in neighboring regions, coordinated actions across borders are suggested for remote units to improve their medical education capacity together. The education sector is suggested to increase quotas for medical universities located in disadvantaged units. It is also the duty of the education sector to plan ahead to match medical student skills with the subtypes of licensed doctors in most need. Third, there is no doubt that the financial sector should keep prompting the influx of the government and private capital into the health labor market. The inflow of non-government capital to support proprietary hospitals also requires partnership between the public and private sectors. That is, the government can give social entrepreneurs tax exemptions or take other measures to encourage building proprietary hospitals while enlarging the recruitment pool of healthcare workers. More importantly, the different focuses of government and social capital make it possible to adjust the densities of certain subtypes of licensed doctors.

## Figures and Tables

**Table 1:** Variable measurements, codes, and descriptions.

Variable	Measurement	Code	Description
Licensed doctor density (LDD)	Clinician density	CD	Number of the clinicians divided by the population and multiplied by 1000
	TCM doctor density	TCMDD	Number of the TCM doctors divided by the population and multiplied by 1000
	Dentist density	DD	Number of the dentists divided by the population and multiplied by 1000
	Public health doctor density	PHDD	Number of the public health doctors divided by the population and multiplied by 1000
	General practitioner density	GPD	Number of the general practitioners divided by the population and multiplied by 1000
Health services demand	Outpatient visits per capita	OV	Number of the outpatient visits divided by the population
	Inpatient visits per capita	IV	Number of the inpatient visits divided by the population
Government health investment	Government health expenditure per capita	GHE	Government health expenditure divided by the population
Social health Investment	Social health expenditure per capita	SHE	Social health expenditure divided by the population
Education capacity	Medical graduate density	MGD	Number of medical graduates divided by the population and multiplied by 1000

**Table 2:** Variables and data resources.

Variables	Research Subjects	Years	Data Resources
Clinician density	31 provincial units	2012–2016	CHSY, CHFPSY, CSY
TCM doctor density	31 provincial units	2012–2016	CHSY, CHFPSY, CSY
Dentist density	31 provincial units	2012–2016	CHSY, CHFPSY, CSY
Public health doctor density	31 provincial units	2012–2016	CHSY, CHFPSY, CSY
General practitioner density	31 provincial units	2012–2016	CHSY, CHFPSY, CSY
Outpatient visits per capita	31 provincial units	2012–2016	CHSY, CHFPSY, CSY
Inpatient visits per capita	31 provincial units	2012–2016	CHSY, CHFPSY, CSY
Government health expenditure per capita	31 provincial units	2012–2016	CHSY, CHFPSY, CSY
Social health expenditure per capita	31 provincial units	2012–2016	CHSY, CHFPSY, CSY
Medical graduate density	31 provincial units	2012–2016	CESY, CSY

**Table 3:** Descriptive statistics of the variables.

Variable	Obs	Mean	Std Err	Min.	Max	Unit
CDD	155	1.640	0.299	0.843	2.802	Person/1000 population
TCMDD	155	0.324	0.109	0.159	0.783	Person/1000 population
DDD	155	0.111	0.058	0.034	0.383	Person/1000 population
PHDD	155	0.089	0.028	0.045	0.193	Person/1000 population
GPD	155	0.120	0.084	0.011	0.404	Person/1000 population
OVPA	155	5.317	1.794	3.007	10.716	Times/person
IVPA	155	0.143	0.031	0.047	0.223	Times/person
GHEPA	155	918.4	363.2	498.2	2584.3	Yuan/person
SHEPA	155	1142.9	839.7	281.2	5739.7	Yuan/person
MGD	155	2.644	1.024	0.491	5.430	Person/10,000 population

Note: Obs = Observation; Std Err = Standard error; Min. = Minimum; Max. = Maximum.

**Table 4:** Best estimations models for estimating different subtypes of licensed doctors.

Variable	Clinicians (SDPM with Spatial Fixed Effects)	TCM Doctors (SLPM with Random Effects)	Dentists (SDPM with Spatial Fixed Effects)	Public Health Doctors (SDPM with Spatial Fixed Effects)	GPs (Non-Spatial Model with Random Effects)
ln(OV)	0.660 *** (0.093)	0.193 ** (0.096)	0.216 (0.135)	0.975 *** (0.183)	0.756 *** (0.211)
ln(IV)	0.275 *** (0.045)	−0.092 (0.056)	−0.011 (0.064)	0.359 *** (0.089)	0.703 *** (0.161)
ln(GHE)	0.082 * (0.048)	0.135 ** (0.054)	0.036 (0.069)	0.263 *** (0.094)	0.302 ** (0.144)
ln(SHE)	0.038 (0.030)	0.157 *** (0.036)	0.223 *** (0.044)	−0.036 (0.060)	0.445 *** (0.117)
ln(MGD)	0.028 (0.019)	−0.006 (0.025)	0.042 (0.028)	0.013 (0.036)	0.013 (0.086)
W × ln(OV)	−0.544 *** (0.158)		−0.122 (0.226)	−0.442 (0.307)	
W × ln(IV)	−0.309 *** (0.089)		0.053 (0.125)	−0.487 *** (0.170)	
W × ln(GHE)	−0.003 (0.076)		0.039 (0.112)	−0.178 (0.148)	
W × ln(SHE)	0.029 (0.058)		0.237 *** (0.088)	−0.084 (0.115)	
W × ln(MGD)	0.053 (0.036)		0.253 *** (0.053)	0.085 (0.070)	
ρ	0.342 *** (0.094)	0.249 *** (0.093)	−0.301 ** (0.125)	0.167 (0.102)	
Log Likelihood	369.2911	222.0605	291.0669	285.3565	
R_w_^2^	0.8740	0.8868	0.9263	0.4521	0.7934
R_b_^2^	0.3642	0.2019	0.5394	0.0588	0.6463
R^2^	0.4095	0.2551	0.5336	0.0647	0.6797
Obs	155	155	155	155	155

Note: Standard error in parentheses, *** *p* < 0.01, ** *p* < 0.05, * *p* < 0.1. W = Spatial Weight Matrix, ln(OV) = ln(Outpatient Visits per capita); ln(IV) = ln(Inpatients visits per capita), ln(GHE) = ln(Government health expenditure per capita), ln(SHE) = ln(Social health expenditure per capita), ln(MGD) = ln(Medical graduates density).

**Table 5:** Direct effects of independent variables on the density of different subtypes of licensed doctors.

Variable	Clinicians	TCM Doctors	Dentists	Public Health Doctors	GPs
OV	0.628 *** (0.090)	0.194 **(0.096)	0.223 (0.141)	0.959 *** (0.178)	0.756 *** (0.211)
IV	0.256 *** (0.047)	−0.094(0.058)	−0.014 (0.067)	0.344 *** (0.090)	0.703 *** (0.161)
GHE	0.085 * (0.045)	0.139 ***(0.052)	0.035 (0.070)	0.260 *** (0.088)	0.302 ** (0.144)
SHE	0.041 (0.031)	0.158 ***(0.037)	0.210 *** (0.047)	−0.041 (0.060)	0.445 *** (0.117)
MGD	0.035 * (0.019)	−0.005(0.026)	0.028 (0.028)	0.018 (0.036)	0.013 (0.086)

Note: *t* statistics in parentheses, *** *p* < 0.01, ** *p* < 0.05, * *p* < 0.1. OV = Outpatient Visits per capita; IV = Inpatients visits per capita, GHE = Government health expenditure per capita, SHE = Social health expenditure per capita, MGD = Ln Medical graduate density.

**Table 6:** Spillover effects of independent variables on the density of different subtypes of licensed doctors.

Variable	Clinicians	TCM Doctors	Dentists	Public Health Doctors	General Practitioners
OV	−0.461 **(0.217)	0.066 (0.051)	−0.155 (0.206)	−0.330 (0.352)	—
IV	−0.306 **(0.125)	−0.032 (0.028)	0.050 (0.101)	−0.489 ** (0.191)	—
GHE	0.030 (0.103)	0.043 * (0.024)	0.019 (0.099)	−0.162 (0.165)	—
SHE	0.063 (0.085)	0.050 ** (0.022)	0.144 * (0.077)	−0.102 (0.133)	—
MGD	0.090 * (0.050)	−0.003 (0.010)	0.200 *** (0.045)	0.103 (0.078)	—

Note: *t* statistics in parentheses, *** *p* < 0.01, ** *p* < 0.05, * *p* < 0.1. “—” means not applicable. OV = Outpatient Visits per capita; IV = Inpatients visits per capita, GHE = Government health expenditure per capita, SHE = Social health expenditure per capita, MGD = Ln Medical graduate density.

## Supplementary Materials

The following are available online at https://www.mdpi.com/1660-4601/16/10/1753/s1, Figure S1: The administrative divisions of China, Table S1: The original data of this study, Table S2: Estimation results of spatial panel econometric models for clinician density, Table S3: Estimation results of spatial panel econometric models for traditional Chinese medicine doctor density, Table S4: Estimation results of spatial panel econometric models for dentist density, Table S5: Estimation results of spatial panel econometric models for public health doctor density, Table S6: Estimation results of spatial panel econometric models for general practitioner density.

## Abbreviations

CD	Clinician density
DD	Dentist density
GHE	Government health expenditure per capita
GP	General practitioner
GPD	General practitioner density
IV	Inpatient visits per capita
LDD	Licensed doctor density
LR	Likelihood Ratio
MGD	Medical graduate density
OLS	Ordinary Least Squares
OV	Outpatient visits per capita
PHDD	Public health doctor density
SDPM	Spatial Durbin Panel Model
SEPM	Spatial Error Panel Model
SHE	Social health expenditure per capita
SLPM	Spatial Lag Panel Model
TCM	Traditional Chinese medicine
TCMDD	TCM doctor density
WHO	World Health Organization

## Appendix A

### Spatial Lag Panel Model

(A1) $$\begin{array}{cc} Ln{\left(LDD\right)}_{it}= & \rho \sum_{j=1}^{n}W_{ij}ln{\left(LDD\right)}_{it}+\beta_{1}ln{\left(OV\right)}_{it}+\beta_{2}ln{\left(IV\right)}_{it}+\beta_{3}ln{\left(GHE\right)}_{it}+\beta_{4}ln{\left(SHE\right)}_{it}\\ & +\beta_{5}ln{\left(MGD\right)}_{it}+\alpha +\mu_{i}+\gamma_{t}+\varepsilon_{it} \end{array}$$

In the SLPM, $W_{ij}$ is a spatial-weighted 31 × 31 matrix (row-standardized) for the 31 geographical units in China, if unit *i* borders unit *j*, then the matrix element $W_{ij}$ = 1, and $W_{ij}$ = 0 otherwise. $W_{ij}ln{\left(LDD\right)}_{it}$ is a vector of the spatial lag (average of the neighbors) of health manpower density; β are the regression coefficients, which are the same as the coefficients in non-spatial models; ρ is a spatial regression coefficient; and $\mu_{i}$ and $\gamma_{t}$ indicate the spatial fixed effects (space-specific, time-invariant) and time-period fixed effects (time-specific, space-invariant), respectively [56].

### Spatial Error Panel Model

In the SEPM, the parameter λ is the spatial autoregressive coefficient, reflecting the influence of the residuals of neighbors on the residuals of a certain area; ε is the spatially autoregressive error term; and the other parameters are the same as for the SLPM.

(A2) $$\begin{array}{c} ln{\left(LDD\right)}_{it}=\beta_{1}ln{\left(OV\right)}_{it}+\beta_{2}ln{\left(IV\right)}_{it}+\beta_{3}ln{\left(GHE\right)}_{it}+\beta_{4}ln{\left(SHE\right)}_{it}+\beta_{5}ln{\left(MGD\right)}_{it}+\alpha +\mu_{i}+\gamma_{t}+\varepsilon_{it}\\ \varepsilon_{it}=\lambda \sum_{j=1}^{n}W_{ij}\varepsilon_{it}+v_{it} \end{array}$$

### Spatial Durbin Panel Model

In the SDPM, $\theta_{1},\theta_{2},\theta_{3},\theta_{4}$ in Formula (A3), as spatial parameters, indicate the impacts of the independent variables in adjacent units on the health manpower density of the target area. The other parameters are the same as for the SLPM and SEPM.

(A3) $$\begin{array}{cc} Ln{\left(LDD\right)}_{it}= & \rho \sum_{j=1}^{n}W_{ij}Ln{\left(LDD\right)}_{it}+\beta_{1}ln{\left(OV\right)}_{it}+\beta_{2}ln{\left(IV\right)}_{it}+\beta_{3}ln{\left(GHE\right)}_{it}+\beta_{4}ln{\left(SHE\right)}_{it}\\ & +\beta_{5}ln{\left(MGD\right)}_{it}+\theta_{1}\sum_{j=1}^{n}W_{ij}ln{\left(OV\right)}_{it}+\theta_{2}\sum_{j=1}^{n}W_{ij}ln{\left(IV\right)}_{it}+\theta_{3}\sum_{j=1}^{n}W_{ij}ln{\left(GHE\right)}_{it}\\ & +\theta_{4}\sum_{j=1}^{n}W_{ij}ln{\left(SHE\right)}_{it}+\theta_{5}\sum_{j=1}^{n}W_{ij}ln{\left(MGD\right)}_{it}+\alpha +\mu_{i}+\gamma_{t}+\varepsilon_{it} \end{array}$$
