# Supplementary material for: Addressing the Licensed Doctor Maldistribution in China: A Demand-And-Supply Perspective
Source: Int J Environ Res Public Health. 2019 May 17;16(10):1753. doi: 10.3390/ijerph16101753 (PMC6571941; doi:10.3390/ijerph16101753)
Supplement: Supplementary file 1 [file ijerph-16-01753-s001.zip › ijerph-486461-supplementary-forxml/Supplementary files/Table S2.docx]

**Table 2.** Estimation results of spatial panel econometric models for clinician density.

| **Variable** | **SDPM with Spatial Fixed Effects**  **(Best Model)** | **SDPM with Time Fixed Effects** | **SDPM with Spatial and Time Fixed Effects** | **SDPM with Random Effects** | **SEPM with Spatial Fixed Effects** | **SLPM with Spatial Fixed Effects** |
| --- | --- | --- | --- | --- | --- | --- |
| **ln(OV)** | 0.660***  (0.093) | −0.043  (0.042) | 0.633 ***  (0.094) | 0.410 ***  (0.088) | 0.518 ***  (0.096) | 0.426 ***  (0.095) |
| **ln(IV)** | 0.275***  (0.045) | 0.151 ***  (0.044) | 0.307 ***  (0.046) | 0.302 ***  (0.042) | 0.332 ***  (0.046) | 0.279 ***  (0.051) |
| **ln(GHE)** | 0.082*  (0.048) | 0.022  (0.038) | 0.125 **  (0.049) | 0.083 *  (0.046) | 0.006  (0.039) | 0.035  (0.045) |
| **ln(SHE)** | 0.038  (0.030) | 0.204 ***  (0.027) | 0.062 **  (0.031) | 0.065 **  (0.032) | 0.041  (0.027) | 0.048 *  (0.029) |
| **ln(MGD)** | 0.028  (0.019) | −0.017  (0.024) | 0.029  (0.018) | 0.032  (0.020) | 0.013  (0.020) | 0.032  (0.021) |
| **W × ln(OV)** | −0.544***  (0.158) | −0.150 **  (0.067) | −0.364  (0.228) | −0.354 ***  (0.129) |  |  |
| **W × ln(IV)** | −0.309***  (0.089) | −0.664 ***  (0.079) | −0.112  (0.120) | −0.345 ***  (0.078) |  |  |
| **W × ln(GHE)** | −0.003  (0.076) | −0.152 **  (0.076) | 0.164  (0.101) | 0.009  (0.073) |  |  |
| **W × ln(SHE)** | 0.029  (0.058) | 0.078  (0.070) | 0.090  (0.069) | 0.007  (0.059) |  |  |
| **W × ln(MGD)** | 0.053  (0.036) | 0.178 ***  (0.045) | 0.105 **  (0.045) | 0.053  (0.036) |  |  |
| $\boldsymbol{\rho}$ | 0.342***  (0.094) | −0.134  (0.114) | 0.191*  (0.112) | 0.330 ***  (0.094) |  | −0.018  (0.089) |
| **λ** |  |  |  |  | 0.441 ***  (0.100) |  |
| **LL** | 369.2911 | 169.3556 | 374.6819 | 273.3824 | 354.9534 | 347.3901 |
| **R_w_^2^** | 0.8740 | 0.1464 | 0.8223 | 0.8676 | 0.8457 | 0.8508 |
| **R_b_^2^** | 0.3642 | 0.7997 | 0.3169 | 0.4467 | 0.2194 | 0.2436 |
| **R^2^** | 0.4095 | 0.7078 | 0.3869 | 0.4977 | 0.2752 | 0.3062 |
| **Obs** | 155 | 155 | 155 | 155 | 155 | 155 |
| **Test** | Hausman test  H0: difference in coefficients not systematic | | | | LR  test | Wald  test |
|  | 𝛘^2^(11) = 37.2 *p* = 0.000 | | | | 𝛘^2^ = 29.6  *p* = 0.000 | 𝛘^2^ = 45.3  *p* = 0.000 |

Note: Standard error in parentheses, *** *p* < 0.01, ***p* < 0.05, * *p* < 0.1.
