# Supplementary material for: Addressing the Licensed Doctor Maldistribution in China: A Demand-And-Supply Perspective
Source: Int J Environ Res Public Health. 2019 May 17;16(10):1753. doi: 10.3390/ijerph16101753 (PMC6571941; doi:10.3390/ijerph16101753)
Supplement: Supplementary file 1 [file ijerph-16-01753-s001.zip › ijerph-486461-supplementary-forxml/Supplementary files/Table S3.docx]

**Table 3.** Estimation results of spatial panel econometric models for traditional Chinese medicine doctor density.

| **Variable** | **SDPM with Spatial Fixed Effects** | **SDPM with Time Fixed Effects** | **SDPM with Spatial and Time Fixed Effects** | **SDPM with Random Effects** | **SEPM with Spatial Random Effects** | **SLPM with Random Effects**  **(Best Model)** |
| --- | --- | --- | --- | --- | --- | --- |
| **ln(OV)** | 0.302 **  (0.121) | 0.135  (0.105) | 0.357 ***  (0.122) | 0.320 ***  (0.110) | 0.180 *  (0.101) | 0.193 **  (0.096) |
| **ln(IV)** | −0.121 **  (0.058) | −0.095  (0.105) | −0.139 **  (0.060) | −0.130 **  (0.056) | −0.079  (0.057) | −0.092  (0.056) |
| **ln(GHE)** | 0.070  (0.062) | 0.237 **  (0.098) | 0.059  (0.063) | 0.101  (0.063) | 0.202 ***  (0.050) | 0.135 **  (0.054) |
| **ln(SHE)** | 0.107 ***  (0.039) | 0.174 ***  (0.066) | 0.112 ***  (0.040) | 0.100 **  (0.041) | 0.186 ***  (0.036) | 0.157 ***  (0.036) |
| **ln(MGD)** | −0.009  (0.024) | −0.015  (0.060) | −0.006  (0.024) | −0.018  (0.026) | 0.006  (0.026) | −0.006  (0.025) |
| **W × ln(OV)** | −0.218  (0.202) | −0.628 ***  (0.163) | 0.206  (0.288) | −0.441 **  (0.181) |  |  |
| **W × ln(IV)** | 0.013  (0.112) | 0.339 *  (0.197) | −0.029  (0.139) | 0.093  (0.105) |  |  |
| **W × ln(GHE)** | 0.009  (0.098) | 0.911 ***  (0.203) | 0.010  (0.122) | 0.028  (0.100) |  |  |
| **W × ln(SHE)** | 0.138 *  (0.078) | 0.074  (0.137) | 0.086  (0.090) | 0.086  (0.081) |  |  |
| **W × ln(MGD)** | 0.064  (0.046) | 0.640 ***  (0.111) | 0.096 *  (0.055) | 0.091  (0.048) |  |  |
| $\boldsymbol{\rho}$ | 0.130  (0.113) | 0.157  (0.125) | −0.007  (0.128) | 0.207 *  (0.109) |  | 0.249 ***  (0.093) |
| **λ** |  |  |  |  | 0.109  (0.132) |  |
| **LL** | 337.9338 | 337.9338 | 337.9338 | 337.9338 | 218.9991 | 222.0605 |
| **R_w_^2^** | 0.8913 | 0.8801 | 0.8826 | 0.8891 | 0.8818 | 0.8868 |
| **R_b_^2^** | 0.2246 | 0.5406 | 0.1142 | 0.3515 | 0.2351 | 0.2019 |
| **R^2^** | 0.2781 | 0.3401 | 0.1556 | 0.3909 | 0.2863 | 0.2551 |
| **Obs** | 155 | 155 | 155 | 155 | 155 | 155 |
| **Test** | Hausman test  H0: difference in coefficients not systematic | | | | LR test | Wald test |
|  | 𝛘^2^(11) = 9.56 *p* = 0.5701 | | | | 𝛘^2^ = 12.0  *p* = 0.035 | 𝛘^2^ = 3.6  *p* = 0.462 |

Note: Standard error in parentheses, *** *p* < 0.01, ** *p* < 0.05, * *p* < 0.1.
