# Supplementary material for: Addressing the Licensed Doctor Maldistribution in China: A Demand-And-Supply Perspective
Source: Int J Environ Res Public Health. 2019 May 17;16(10):1753. doi: 10.3390/ijerph16101753 (PMC6571941; doi:10.3390/ijerph16101753)
Supplement: Supplementary file 1 [file ijerph-16-01753-s001.zip › ijerph-486461-supplementary-forxml/Supplementary files/Table S4.docx]

**Table 4.** Estimation results of spatial panel econometric models for dentist density.

| **Variable** | **SDPM with Spatial Fixed Effects** | **SDPM with Time Fixed Effects** | **SDPM with Spatial and Time Fixed Effects** | **SDPM with Random Effects** | **SEPM with Spatial Fixed Effects** | **SLPM with Spatial Fixed Effects** |
| --- | --- | --- | --- | --- | --- | --- |
| **ln(OV)** | 0.216  (0.135) | 0.405 ***  (0.086) | 0.180  (0.136) | 0.317 ***  (0.133) | 0.177  (0.117) | 0.184  (0.127) |
| **ln(IV)** | −0.011  (0.064) | −0.301 ***  (0.091) | −0.032  (0.067) | −0.035  (0.066) | 0.044  (0.067) | 0.040  (0.069) |
| **ln(GHE)** | 0.036  (0.069) | 0.046  (0.075) | −0.005  (0.071) | 0.031  (0.074) | 0.123 **  (0.053) | 0.118 *  (0.063) |
| **ln(SHE)** | 0.223 ***  (0.044) | 0.644 ***  (0.053) | 0.206 ***  (0.044) | 0.260 ***  (0.049) | 0.321 ***  (0.037) | 0.301 ***  (0.042) |
| **ln(MGD)** | 0.042  (0.028) | 0.205 ***  (0.047) | 0.051 *  (0.028) | 0.036  (0.030) | 0.095 ***  (0.030) | 0.057 *  (0.029) |
| **W × ln(OV)** | −0.122  (0.226) | −0.938 ***  (0.141) | −0.473  (0.298) | −0.206  (0.221) |  |  |
| **W × ln(IV)** | 0.053  (0.125) | −1.541 ***  (0.162) | −0.082  (0.154) | −0.082  (0.128) |  |  |
| **W × ln(GHE)** | 0.039  (0.112) | −0.944 ***  (0.155) | −0.121  (0.136) | −0.040  (0.117) |  |  |
| **W × ln(SHE)** | 0.237 ***  (0.088) | −0.158  (0.160) | 0.263 ***  (0.101) | 0.221 **  (0.097) |  |  |
| **W × ln(MGD)** | 0.253 ***  (0.053) | −0.180 **  (0.089) | 0.175 ***  (0.062) | 0.208 ***  (0.058) |  |  |
| $\boldsymbol{\rho}$ | −0.301 **  (0.125) | 0.060  (0.110) | −0.377 ***  (0.127) | −0.093  (0.125) |  | 0.063  (0.089) |
| **λ** |  |  |  |  | −0.274 *  (0.143) |  |
| **LL** | 291.0669 | 291.0669 | 291.0669 | 291.0669 | 300.4646 | 298.9125 |
| **R_w_^2^** | 0.9263 | 0.7547 | 0.9062 | 0.9254 | 0.9136 | 0.9156 |
| **R_b_^2^** | 0.5394 | 0.8886 | 0.6719 | 0.5896 | 0.5767 | 0.5481 |
| **R^2^** | 0.5336 | 0.4860 | 0.6767 | 0.5978 | 0.5752 | 0.5518 |
| **Obs** | 155 | 155 | 155 | 155 | 155 | 155 |
| **Test** | Hausman test  H0: difference in coefficients not systematic | | | | LR test | Wald test |
|  | 𝛘^2^(11) = 25.4 *p* = 0.008 | | | | 𝛘^2^ = 26.7  *p* = 0.000 | 𝛘^2^ = 10.6  *p* = 0.031 |

Note: Standard error in parentheses, *** *p* < 0.01, ** *p* < 0.05, * *p* < 0.1.
