# Supplementary material for: Addressing the Licensed Doctor Maldistribution in China: A Demand-And-Supply Perspective
Source: Int J Environ Res Public Health. 2019 May 17;16(10):1753. doi: 10.3390/ijerph16101753 (PMC6571941; doi:10.3390/ijerph16101753)
Supplement: Supplementary file 1 [file ijerph-16-01753-s001.zip › ijerph-486461-supplementary-forxml/Supplementary files/Table S5.docx]

**Table 5.** Estimation results of spatial panel econometric models for public health doctor density.

| **Variable** | **SDPM with Spatial Fixed Effects**  **(Best Model)** | **SDPM with Time Fixed Effects** | **SDPM with Spatial and Time Fixed Effects** | **SDPM with Random Effects** | **SEPM with Spatial Fixed Effects** | **SLPM with Spatial Fixed Effects** |
| --- | --- | --- | --- | --- | --- | --- |
| **ln(OV)** | 0.975 ***  (0.183) | −0.127  (0.098) | 0.913 ***  (0.183) | 0.671 ***  (0.170) | 0.636 ***  (0.184) | 0.656 ***  (0.186) |
| **ln(IV)** | 0.359 ***  (0.089) | −0.181 *  (0.100) | 0.395 ***  (0.091) | 0.343 ***  (0.085) | 0.432 ***  (0.101) | 0.375 ***  (0.100) |
| **ln(GHE)** | 0.263 ***  (0.094) | 0.249 ***  (0.094) | 0.305 ***  (0.095) | 0.361 ***  (0.098) | −0.079  (0.079) | −0.065  (0.079) |
| **ln(SHE)** | −0.036  (0.060) | 0.292 ***  (0.063) | −0.014  (0.060) | −0.011  (0.063) | −0.152 ***  (0.055) | −0.157 ***  (0.055) |
| **ln(MGD)** | 0.013  (0.036) | −0.241 ***  (0.055) | 0.016  (0.036) | −0.008  (0.039) | −0.012  (0.041) | 0.003  (0.039) |
| **W × ln(OV)** | −0.442  (0.307) | 0.011  (0.160) | −0.538  (0.406) | −0.296  (0.262) |  |  |
| **W × ln(IV)** | −0.487 ***  (0.170) | −0.684 ***  (0.185) | −0.237  (0.218) | −0.470 ***  (0.154) |  |  |
| **W × ln(GHE)** | −0.178  (0.148) | −0.724 ***  (0.180) | 0.053  (0.195) | −0.185  (0.149) |  |  |
| **W × ln(SHE)** | −0.084  (0.115) | −0.176  (0.148) | 0.068  (0.134) | −0.139  (0.118) |  |  |
| **W × ln(MGD)** | 0.085  (0.070) | −0.272 **  (0.115) | 0.099  (0.083) | 0.076  (0.072) |  |  |
| $\boldsymbol{\rho}$ | 0.167  (0.102) | −0.105  (0.129) | −0.041  (0.124) | 0.198 *  (0.103) |  | −0.042  (0.107) |
| **λ** |  |  |  |  | 0.160  (0.116) |  |
| **LL** | 285.3565 | 285.3565 | 285.3565 | 285.3565 | 246.2362 | 245.3993 |
| **R_w_^2^** | 0.4521 | 0.0053 | 0.0868 | 0.4314 | 0.2926 | 0.2993 |
| **R_b_^2^** | 0.0588 | 0.6268 | 0.0708 | 0.1202 | 0.0486 | 0.0425 |
| **R^2^** | 0.0647 | 0.4018 | 0.0691 | 0.1271 | 0.0369 | 0.0321 |
| **Obs** | 155 | 155 | 155 | 155 | 155 | 155 |
| **Test** | Hausman test  H0: difference in coefficients not systematic | | | | LR test | Wald test |
|  | 𝛘2(11) = 42.9 *p* = 0.000 | | | | 𝛘^2^ = 36.9  *p* = 0.000 | 𝛘^2^ = 26.1  *p* = 0.000 |

Note: Standard error in parentheses, *** *p* < 0.01, ** *p* < 0.05, **p* < 0.1.
