# Supplementary material for: Addressing the Licensed Doctor Maldistribution in China: A Demand-And-Supply Perspective
Source: Int J Environ Res Public Health. 2019 May 17;16(10):1753. doi: 10.3390/ijerph16101753 (PMC6571941; doi:10.3390/ijerph16101753)
Supplement: Supplementary file 1 [file ijerph-16-01753-s001.zip › ijerph-486461-supplementary-forxml/Supplementary files/Table S6.docx]

**Table 6.** Estimation results of spatial panel econometric models for general practitioner density.

| **Variable** | **SDPM with Spatial Fixed Effects** | **SDPM with Time Fixed Effects** | **SDPM with Spatial and Time Fixed Effects** | **SDPM with Random Effects** | **SEPM with Random Effects** | **SLPM with Random Effects** | **Non−Spatial Model with Random Effects**  **(Best Model)** |
| --- | --- | --- | --- | --- | --- | --- | --- |
| **ln(OV)** | 0.715  (0.499) | 0.326 **  (0.138) | −0.213  (0.441) | 0.515 **  (0.253) | 0.558 ***  (0.154) | 0.565 ***  (0.153) | 0.756 ***  (0.211) |
| **ln(IV)** | 0.525 **  (0.238) | 0.728 ***  (0.142) | 0.738 ***  (0.216) | 0.693 ***  (0.175) | 0.303 ***  (0.102) | 0.266 ***  (0.097) | 0.703 ***  (0.161) |
| **ln(GHE)** | 1.001 ***  (0.257) | −0.347 ***  (0.127) | 0.890 ***  (0.226) | 0.260  (0.208) | 0.010  (0.083) | 0.015  (0.082) | 0.302 **  (0.144) |
| **ln(SHE)** | 0.307 *  (0.163) | 0.529 ***  (0.091) | 0.228  (0.143) | 0.415 ***  (0.135) | −0.141 **  (0.059) | −0.145 **  (0.058) | 0.445 ***  (0.117) |
| **ln(MGD)** | −0.108  (0.099) | −0.308 ***  (0.077) | −0.101  (0.085) | −0.052  (0.094) | −0.058  (0.045) | −0.044  (0.043) | 0.013  (0.086) |
| **W × ln(OV)** | 0.812  (0.883) | 0.389 *  (0.222) | −3.292 ***  (0.953) | 0.610  (0.449) |  |  |  |
| **W × ln(IV)** | 0.787  (0.480) | −0.604 **  (0.272) | 1.340 **  (0.521) | −0.274  (0.331) |  |  |  |
| **W × ln(GHE)** | 0.487  (0.429) | 0.167  (0.259) | 0.483  (0.453) | 0.363  (0.313) |  |  |  |
| **W × ln(SHE)** | −1.086 ***  (0.322) | 0.645 ***  (0.216) | −0.380  (0.317) | −0.322  (0.265) |  |  |  |
| **W × ln(MGD)** | 0.528 ***  (0.197) | 0.219  (0.154) | 0.024  (0.196) | 0.344 **  (0.167) |  |  |  |
| $\boldsymbol{\rho}$ | −0.059  (0.113) | −0.295 **  (0.133) | −0.523 ***  (0.121) | 0.077  (0.104) |  | −0.003  (0.109) |  |
| **λ** |  |  |  |  | 0.139  (0.137) |  |  |
| **LL** | 227.938 | 227.938 | 227.938 | 37.152 | 146.344 | 145.839 |  |
| **R_w_^2^** | 0.8220 | 0.7354 | 0.7204 | 0.8043 | 0.2739 | 0.2802 | 0.7934 |
| **R_b_^2^** | 0.2752 | 0.7602 | 0.0434 | 0.6558 | 0.0022 | 0.0021 | 0.6463 |
| **R^2^** | 0.3682 | 0.7536 | 0.0073 | 0.6898 | 0.0006 | 0.0006 | 0.6797 |
| **Obs** | 155 | 155 | 155 | 155 | 155 | 155 | 155 |
| **Test** | Hausman test  H0: difference in coefficients not systematic | | | | LR test | Wald test | Hausman test |
|  | 𝛘^2^(11) = 12.0 *p* = 0.368 | | | | 𝛘^2^ = 5.9  *p* = 0.318 | 𝛘^2^ = 6.1  *p* = 0.196 | 𝛘^2^(5) = 1.5  *p* = 0.909 |

Note: Standard error in parentheses, *** *p* < 0.01, ** *p* < 0.05, * *p* < 0.1.
