# Supplementary figures and images for: Addressing the Licensed Doctor Maldistribution in China: A Demand-And-Supply Perspective
Source: Int J Environ Res Public Health. 2019 May 17;16(10):1753. doi: 10.3390/ijerph16101753 (PMC6571941; doi:10.3390/ijerph16101753)

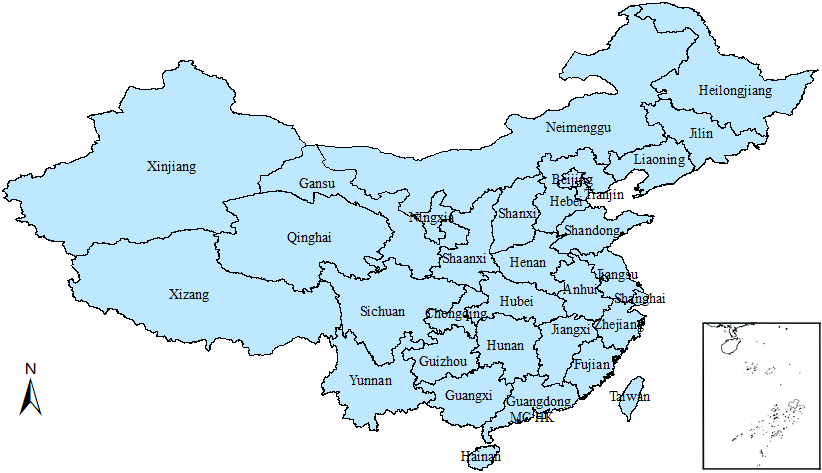


**Figure 1.** The administrative divisions of China.

Supplement: Supplementary file 1 [file ijerph-16-01753-s001.zip › ijerph-486461-supplementary-forxml/Supplementary files/Figure S1.docx]
